# Event-Specific Transmission Forecasting of SARS-CoV-2 in a Mixed-Mode Ventilated Office Room Using an ANN

**DOI:** 10.3390/ijerph192416862

**Published:** 2022-12-15

**Authors:** Nishant Raj Kapoor, Ashok Kumar, Anuj Kumar, Dilovan Asaad Zebari, Krishna Kumar, Mazin Abed Mohammed, Alaa S. Al-Waisy, Marwan Ali Albahar

**Affiliations:** 1 Academy of Scientific and Innovative Research (AcSIR), Ghaziabad 201002, India; 2 Architecture and Planning Department, CSIR-Central Building Research Institute, Roorkee 247667, India; 3 Building Energy Efficiency Division, CSIR-Central Building Research Institute, Roorkee 247667, India; 4 Department of Computer Science, College of Science, Nawroz University, Duhok 42001, Iraq; 5 Department of Hydro and Renewable Energy, Indian Institute of Technology, Roorkee 247667, India; 6 College of Computer Science and Information Technology, University of Anbar, Anbar 31001, Iraq; 7 Computer Technologies Engineering Department, Information Technology College, Imam Ja’afar Al-Sadiq University, Baghdad 10064, Iraq; 8 School of Computer Science, Umm Al-Qura University, Mecca 24382, Saudi Arabia

**Keywords:** artificial neural network, SARS-CoV-2, carbon dioxide concentration, public health, real-time monitoring, mixed-mode ventilation, office environment, air-conditioned buildings

## Abstract

The emerging novel variants and re-merging old variants of SARS-CoV-2 make it critical to study the transmission probability in mixed-mode ventilated office environments. Artificial neural network (ANN) and curve fitting (CF) models were created to forecast the R-Event. The R-Event is defined as the anticipated number of new infections that develop in particular events occurring over the course of time in any defined space. In the spring and summer of 2022, real-time data for an office environment were collected in India in a mixed-mode ventilated office space in a composite climate. The performances of the proposed CF and ANN models were compared with respect to traditional statistical indicators, such as the correlation coefficient, *RMSE*, *MAE*, *MAPE*, *NS* index, and a20-index, in order to determine the merit of the two approaches. Thirteen input features, namely the indoor temperature (*T_In_*), indoor relative humidity (*RH_In_*), area of opening (*A_O_*), number of occupants (*O*), area per person (*A_P_*), volume per person (*V_P_*), CO_2_ concentration (*CO*_2_), air quality index (*AQI*), outer wind speed (*W_S_*), outdoor temperature (*T_Out_*), outdoor humidity (*RH_Out_*), fan air speed (*F_S_*), and air conditioning (*AC*), were selected to forecast the R-Event as the target. The main objective was to determine the relationship between the CO_2_ level and R-Event, ultimately producing a model for forecasting infections in office building environments. The correlation coefficients for the CF and ANN models in this case study were 0.7439 and 0.9999, respectively. This demonstrates that the ANN model is more accurate in R-Event prediction than the curve fitting model. The results show that the proposed ANN model is reliable and significantly accurate in forecasting the *R-Event* values for mixed-mode ventilated offices.

## 1. Introduction

Since prehistoric times, infectious illnesses have impacted several civilizations. There have been several devastating viral outbreaks throughout the past century. Severe acute respiratory syndrome coronavirus” (SARS-CoV) (2002), influenza A virus subtype H1N1 (A/H1N1) (2009), Middle East respiratory syndrome coronavirus (MERS-CoV) (2012), Ebola virus (2013), and severe acute respiratory syndrome coronavirus-2 (SARS-CoV-2) are a few of the deadliest viral pandemics that have had widespread effects in the past 20 years (2002–present) [1]. Millions of people have lost their lives as a result of these pandemics in the last two decades, and many more are currently suffering. Many more viral outbreaks are anticipated in the not too distant future as well. Coronavirus disease is referred to as COVID-19, and the two numeric characters at the end stand for the genesis year of this virus, which is 2019. The coronavirus has been spreading quickly over the globe since the start of 2020. Several countries are anticipating the emergence of a new wave of COVID-19, even though many of them have already suffered the severe consequences of previous waves. Throughout this study, SARS-CoV-2 is referred to as “SC-2”. Every country has suffered significant omnidirectional losses as a result of this SC-2 pandemic. The loss of human life is without a doubt the greatest loss. According to the World Health Organization (WHO) [2], as of October 2022, COVID-19 has infected over 600 million people globally, causing over 6.5 million deaths.

Currently, there are three identified SC-2 transmission modes. Contact transmission and droplet transmission are the two most well-known of the three modes. Later research studies performed on rapid transmission revealed the third mode of infectious transmission, namely through inhaling microscopic airborne droplet nuclei (aerosols) [3]. Different sized droplets are produced when an infected individual breathes, speaks, sneezes, and coughs; the bigger droplets quickly settle in the 1–2 m distance, according to a number of recent studies. Droplets with a smaller diameter can travel far and land more than two meters from the source of the virus. Greater viral concentrations are carried by the smaller particles, which may move up to 6 m owing to air flow and the lack of gravitational pull [4,5,6]. The primary reason for the unprecedented infectious spread rate is airborne transmission, and this is backed up by several research studies conducted throughout the world. Viral transmission issues have received more attention in closed structures, such as hospitals, hotels, commercial buildings, schools, offices, as well as other buildings and structures, in order to maintain health, productivity, and economic growth. It is generally known that the majority of “super-spreading” incidents, in which multiple persons are discovered to be infected, take place in closed structures, including workplaces, restaurants, schools, houses, apartment buildings, hospitals, and meeting rooms [7,8,9,10,11,12,13]. Most of time is spent indoors by the majority of the population, and the status of their indoor environment directly affects how they perceive comfort. Pathogen transmission inside closed structures is also nudged by IEQ. It has been established that SC-2 can be transmitted through the air, which accounts for the sharp rise of COVID-19 cases in buildings with inadequate ventilation [14,15,16,17]. Additionally, anthropogenic respiratory activities have an impact on the transmission rate, since there are fluctuations in the number, speed, size, and settling distance of respirationally ejected pathogenic particles, as well as in the frequency and length of activity occurrence. The most frequent respiratory actions in office settings include breathing, talking, coughing, and sneezing.

In virtual office environments, Shrestha et al. [18] investigated the aerosol dispersion of SC-2 and found that unventilated areas are more likely to transmit diseases. They concluded that even with minimal occupancy, a building with inadequate ventilation can result in a considerable aerosol buildup. In addition to SC-2, poor ventilation is also related to an increase in the prevalence of other respiratory ailments. However, in accordance with the present emphasis on SC-2, researchers have noted that poor ventilation is a significant contributor in the transmission of SC-2 within various building types. Numerous scholars, decision-makers, building-related research organizations, and HVAC societies, as well as associations from various nations, have published studies on the management and operation of ventilation systems during pandemics. The WHO has issued recommendations and guidelines to combat COVID-19, along with a number of other countries throughout the globe, including the United States, European Union, Canada, India, China, and Japan [19,20,21,22]. Almost all of the recommendations make it seem desirable to boost exterior air ventilation in a building throughout the day.

One of the most fundamental techniques to consume less energy with significant air changes and better comfort conditions in buildings is mixed-mode (MM) ventilation. Significant air changes per hour are achievable with MM ventilation at low operating costs and efficient heat removal to create comfortable indoor environment. Both natural forces and the use of mechanical equipment such as fans, air conditioners, and coolers produce airflow through the building with MM ventilation. The buoyant forces produced by temperature fluctuations inside the building, wind from the outside, and the fan speed inside the building, as well as all other operating ventilation and air conditioning equipment are necessary for MM ventilation. Buildings may be designed and operated to benefit from the interactions of these natural forces with the aid of mechanical equipment. According to a previous study [23], the period of aerosol dispersion is multiplied ten-fold in a room with poor ventilation.

The indoor ventilation and carbon dioxide (CO_2_) concentration have a particular connection in a steady-state situation [24]. As a result, the indoor concentration of CO_2_ might be used as a proxy indicator if an appropriate instrument to monitor indoor ventilation is unavailable. The interior CO_2_ levels indicate the ventilation effectiveness of a building in connection to air changes, mechanical devices, air flow patterns (depending on internal and external environments, human activities indoors), opening areas, and the occupant density, as well as the spatial volume of occupied space [25]. Traditional CO_2_ monitoring methods do not capture spatiotemporal fluctuations in CO_2_ concentration levels. Recent technical advancements in CO_2_ monitoring and prediction have allowed for reliable predictions of CO_2_ concentration levels within buildings. These advancements further provide information on individuals’ CO_2_ exposure. Furthermore, the number of accurate air changes per hour (ACH) is difficult to discover in an actual office environment, since the circumstances in mixed-mode ventilated building spaces are very dynamic. The CO_2_ concentration in standard indoor environments must be maintained below 1000 ppm. This should be prioritized for improvement if the concentration exceeds 1500 ppm or above. As per the SAGE-EMG recommendations for COVID-19 circumstances [26], the CO_2_ levels in buildings where a lot of aerosols are created should be kept below 800 ppm. CO_2_ might be utilized as a surrogate to identify the spread of infection caused by SC-2, since both the COVID-19 spread and CO_2_ concentration are affected by the occupant density in any enclosed environment. 

Based on the carbon dioxide content, Rudnick and Milton [27] assessed the chances in viral transmission through air indoors. They created a model that uses the CO_2_ content as a gauge for respired breath exposure, as well as determining how much of the air being breathed has previously been expelled by a dweller within the structure. The scientists developed a CO_2_-based risk model without considering the outer air supply rate or assuming that it remains constant over time. They also avoided assuming that the concentration of an infectious agent had reached the steady state. Likewise, Peng and Jimenez [28] suggested evaluating the CO_2_ levels as a SC-2 infection risk proxy for various interior locations and activities in 2021. In a range of typical indoor environments, the authors developed analytical formulas for CO_2_-based risk proxies. The authors cited a restriction whereby the estimations of infection risk, which are mostly dependent on viral exhalation rates, have significant uncertainties. Nonetheless, they advised installing low-cost risk monitoring systems based on CO_2_ sensors to check for indoor infection in order to advance public safety and health. To reduce COVID-19 transmission, Bazant and Bush [29] advised limiting the amount of time spent in mutual places with an infected person. Later, Bazant et al. [30] recast the safety advice in terms of the mean exhaled CO_2_ concentration and occupancy time in an indoor setting, permitting the use of CO_2_ sensors in the risk evaluation of aerial viral transmission. Based on monitored CO_2_ data, the authors created a mathematical model to estimate the likelihood of aerial transmission. The authors came to the firm conclusion that while CO_2_ levels are regarded as a direct signal for ventilation and air mixing, they may also be utilized to evaluate the risk of SC-2 viral aerial transmission. A review on the aerial transmission of respiratory viruses was provided by Wang et al. [31]. By serving as indicators of the buildup of exhaled air, CO_2_ sensors may be used to track ventilation and enhance it. It has been suggested that CO_2_ levels be kept between 700 and 800 ppm, however the method of ventilation should also be taken into account. “ArchABM” is a brand-new agent-based simulator that Martinez et al. [32] created. By estimating ventilation characteristics and adequate room sizes, as well as evaluating the impacts of policies while accounting for IAQ as a consequence of complex building–human interaction patterns, the system is made to help with either the creation of brand new structures or the modification of already existing ones. In this study, an aerosol model that had already been published was updated to calculate the time-dependent CO_2_ and viral quanta concentrations in each room and the inhaled CO_2_ and viral quanta concentrations for every dweller for a day as a gauge of the physiological response. Recently, Kapoor et al. [33,34] published a couple of studies to predict AI-based event-specific airborne viral transmission in a naturally ventilated office room. In [33], the authors connected IEQ parameters (CO_2_ levels, temperature, and humidity) with the occupancy rate, occupant behavior of door and window opening, outdoor pollution level, air conditioning with an operable fan, and event-specific viral transmission probability via ANN and curve fitting methods. In another study [34], the abovementioned parameters were connected using various other machine learning (ML) methods. This study advances existing studies by incorporating AI to forecast the aerial transmission by means of data on the indoor CO_2_ concentration, occupancy rate, occupant behavior, and other indoor and outdoor environmental factors that are acquired in real-time mixed-mode ventilated office environments.

**Figure 1:**
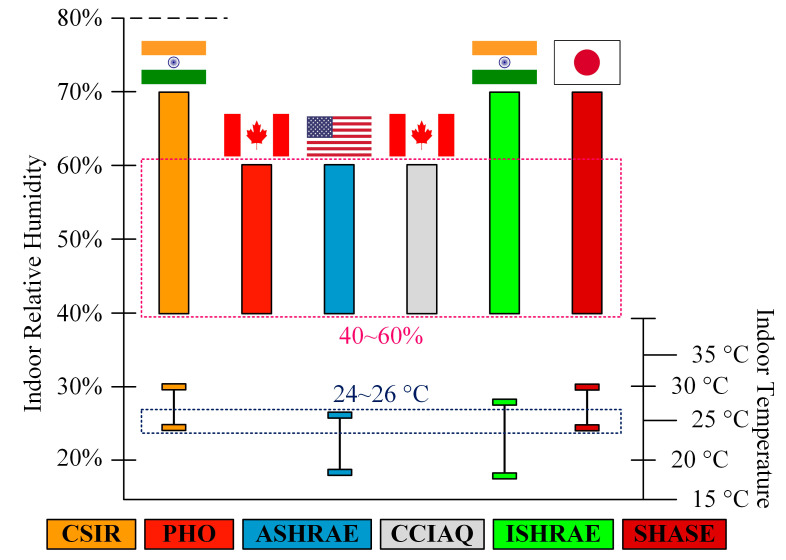
Indoor air temperature and relative humidity ranges for safe–comfortable indoor environments.

The temperature and humidity are also crucial indoor environmental factors that influence viral survival. The temperature and humidity both have a significant impact on indoor human comfort and have a direct impact on how people feel. Mecenas et al. [35] conducted a review of the literature on the impacts of temperature and humidity on COVID-19 transmission and concluded that future research should take these factors into consideration because they may have an impact on the disease’s spread. The relative humidity and temperature were also identified as essential factors in a number of international guidelines. Many of these guidelines suggested boundary conditions for indoor temperature as well as relative humidity, which took into account both indoor human comfort and the transmission of SC-2. Figure 1 represents the boundary conditions recommended by several guidelines for the indoor air temperature and relative humidity to prevent the transmission of SC-2 while maintaining comfort [20,22,36,37,38,39,40,41]. The common safe–comfortable temperature range among the mentioned guidelines is from 24 °C to 26 °C and the safe–comfortable indoor relative humidity range is from 40% to 60%. 

Other crucial factors for minimizing SC-2 transmission in a closed building include the occupant behavior, outside environmental conditions, and indoor occupant density [42,43]. The guidelines on COVID-19-appropriate behavior in office contexts have been released by a number of organizations and governments from across the world [44,45]. Some of the key suggestions made to employers for a safe workplace were maintaining reduced occupancy, good hygiene, sufficient aeration, appropriate safety measures, timely training schedules, accurate information flows, quick responses to any kind of illness or symptom, and worker health monitoring. 

The chance of SC-2 spreading in an office setting can be significantly reduced by using an appropriate door and window opening strategy in accordance with spatiotemporal variation and occupant activity behavior patterns, as well as comfort. Almost all of the recommendations call for opening windows and doors to improve ventilation and reduce stale air generated within by exhalation. Opening windows or doors, however, is not always a smart option. Particularly in situations where there is unwanted excessive light, high levels of outside pollution entering the building (based on *AQI* measurements), visual privacy concerns, fly and insect invasion risks, uncomfortable air movements or drafts, or excessive outside noise, which can affect user concentration an in turn increase errors, leading to productivity losses. However, using mechanical devices combined with altered ratios of door and window opening operations can solve these problems up to a certain level. MM ventilation can be an effective solution in the abovementioned situations against viral transmission problems. Ceiling fans, exhaust fans, air conditioners, coolers, and table fans can be used effectively to ventilate the indoor spaces containing operable doors and windows. The additional mechanical components merged with natural ventilation help in reducing the viral transmission if used properly.

The most significant components of the air quality index, or *AQI*, are the PM_10_ and PM_2.5_, which indicate the levels of pollutants [46]. Several researchers have tried to find the relation between the particulate matter in buildings and SC-2 transmission [47,48,49,50,51]. Since the size of the virus is very small, it can easily settle on dust and particulate matter and then be transmitted by the resuspension of those particles inside closed buildings due to human activities or natural forces. As the gravitational force is negligible on the dry nucleus form of virus, the SC-2 virus can survive longer in the air and on surfaces; this makes it a dangerous viral form. The chances of inhaling the free floating viral load in the air is much higher in closed environments with higher occupancy rates. In addition to this, the minuscule pathogens can penetrate deeply in our bodies and create severe problems. Most of the features presented above are correlated, with some having strong relationships while others have weaker linkages. The diurnal trends and associated variations among indoor environmental parameters have been studied by researchers globally [52,53,54].

In connection to SC-2, Tupper et al. [55] developed the idea of “*Event-R*”. The *Event-R* rate is the “expected number of new infections due to the presence of a single infectious individual at an event”, according to Tupper et al. The authors discovered a basic association between the “*Event-R*” rate and four different characteristics, namely the spread intensity, degree of mixing, individual proximity, and exposure time. The *R-Event* is another term for the *Event-R*, and either can be used in real time. In addition to the infection probability, the *R-Event* was also employed by REHVA in their revised (Version 2.1) airborne COVID-19 prediction tool [56].

The precise infection probability is now being determined by scientific research on the antecedent transmission of several SC-2 virus strains in indoor environments around the world. In bounded spaces, the scientific community examines two distinct sorts of infection probability: (i) based on the CO_2_ exhaled by the enclosed space occupiers and (ii) based on the air change rate. The first type is coupled with on-site dynamic spatiotemporal interior and outside environmental data to forecast the *R-Event*. The *R-Event* is a more advanced and informative method for infection probability. The *R-Event* value is the multiplication of the infection probability by the ratio of susceptible individuals to the infected person; alternative values of the *R-Event* can be provided for different occupancy rates, whereas the infection probability remains the same regardless of the occupancy rate. The REHVA calculator (v2.1) is used in determining the *R-Event* under mixed-mode settings and recording it synchronously with other real-time information to create a relationship between indoor CO_2_ levels and *R-Event* values. To determine the likelihood of airborne transmission, an unknown solitary contaminated person (among four static individuals inside the case office room) is taken as an infection source (through aerosols only). The purpose of this study is to assist readers in reliably predicting the *R-Event* value via indoor CO_2_ levels in an office area. In addition to CO_2_, the interior and exterior environmental characteristics, occupancy rate, and occupant information are essential for the prediction results.

If an SC-2-contaminated sick occupant is occupying a closed indoor space with other susceptible occupants and all are inhaling and exhaling simultaneously, then high occupant density and lower ventilation rates can lead to an accumulation of CO_2_ with freely suspended SC-2 virus (in air) particles that are liable for the aerial transmission of SC-2. To forecast the probability of infection using objective and subjective data, many numerical models have been established, and there have been several simulation studies as well. As noted in the introduction, only a few scientists have tried to connect the dots between indoor CO_2_ concentration and SC-2 transmission rates. A model based on sophisticated computing techniques such as artificial intelligence (AI) has yet to be presented in a real-time research study. 

There is less research on the relationship between indoor CO_2_ levels and viral transmission [27,28,29,30,31,32,33,34]. However, as CO_2_ may be utilized scientifically as a proxy for indoor viral transmission, similarly the indoor CO_2_ concentration can be used to predict the probable number of infected people as well. In practical dynamic environments such as workplaces and classrooms, the CO_2_ levels are not linked to the amount of infectious pathogens present in the air. 

In this study, as a proxy for the chance of SC-2 transmission, the *R-Event* value was predicted using a supervised ML-based ANN model, as well as a curve fitting (CF) model. Another advantage of this study was the development of the CO_2_ concentration database and investigation of the occupants’ exposure to indoor CO_2_ by mapping the office staff members’ (subjects) activities within the MM ventilated office room and combining them with the *R-Event*. The three research objectives of this work were as follows:

1. Gathering real-time spatiotemporal data for both subjective (occupancy-related) and objective (environmental) variables in office environment to use machine learning (ML) techniques to create links among the parameters;
2. Creating a unique relationship between the *R-Event* (event-specific infection probability) value and CO_2_ concentration in mixed-mode ventilated office environments;
3. Comparing novel CF and ANN models (developed for a mixed-mode office environment) for the prediction of *R-Event* values.

The rest of this study is organized as follows. Section 2 explains the novelty and objectives of the study. Section 3 deals with the materials and methods used in this research, including the collection, standardization, and filtration of the data. Section 4 defines the CF method and ANN technique in detail. Section 5 discusses the results of the ANN models and the CF model with the ANN formulation. The last section, Section 6, concludes the work done in this study with the limitations of this research and potential scope for future work.

## 2. Materials and Methods

CO_2_ was continually monitored in an office using an EXTECH Indoor Air Quality Meter/Data-Logger Model EA80. The EA80 instrument measures CO_2_ concentrations using a maintenance-free CO_2_ sensor with a 0 to 6000 ppm range. The equipment was maintained away from the static occupants because CO_2_ being directly emitted over the instrument degrades the measurement accuracy. The EXTECH Heat Stress WBGT Meter Model HT200 was used to detect the temperature and humidity. Both devices were installed at 0.8 m above the tile floor. Table 1 and Table 2 give the technical specifications for both devices. Both factory-calibrated instruments were examined before usage. Alternative equipment models with higher accuracy are also available for the same purpose; however, owing to our financial restrictions, these models were utilized throughout this investigation. The *AQI* was recorded from [46]. The calculations were carried out on a desktop computer using MATLAB R2021a.

The prediction model was built using 2207 datasets out of a total of 2640 datasets. This study included measurements of the indoor temperature (*T_In_*), indoor relative humidity (*RH_In_*), area of opening (*A_O_*), number of occupants (*O*), area per person (*A_P_*), volume per person (*V_P_*), CO_2_ concentration (CO_2_), air quality index (*AQI*), outer wind speed (*W_S_*), outdoor temperature (*T_Out_*), outdoor humidity (*RH_Out_*), fan air speed (*F_S_*), air conditioning (*AC*), and one target variable, the Event-R (*R-Event*). The datasets were chosen based on the likelihood of having at least two occupants present in the workplace. This study did not take into account single-occupancy datasets. Furthermore, datasets in which no mechanical device was engaged were not considered. As a result, 433 datasets were removed from the total collected datasets for mixed-mode ventilated office environments. 

The occupancy rate of the office space has the most impact on the CO_2_ concentration, since the occupants are the major source of emissions. The output *R-Event* is the anticipated number of new infections that arise in any event occurring during a time period “*T*” in any enclosed environment. Equation (1) presents the mathematical form of the *R-Event* [55]. However, the *R-Event* is calculated mathematically using the SC-2 infection probability calculator in REHVA version 2.1. Equation (2) is the REHVA-calculator-based mathematical formulation for the *R-Event* [56]. The surroundings of the MM ventilated office building have an impact on the conditions inside as well. Office buildings close to busy roads, markets, and densely populated areas are frequently seen to be affected by environmental factors. As a result, this study also takes external environmental variables into account.

(1) $$REvent=\frac{kT}{t}(1-e^{-\beta t}),$$

“*T*” represents the length of the entire event; “*t*” represents the length of time that a solitary susceptible occupant made contact with an infected occupant; “*β*” denotes the probability of transmission, which is constant with respect to time; “1 − *e^−βt^*” denotes the likelihood that any susceptible occupant will become infected; “*k*” denotes the susceptible occupants (total) who made contact with the infectious person, which may be expressed simply as Equation (2):

(2) $$REvent=\frac{Infection\;probability\times Number\;of\;susceptible\;person}{Number\;of\;infectitious\;person},$$

**Figure 2:**
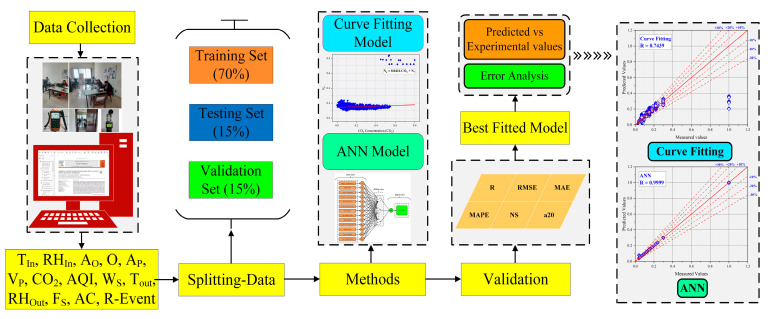
Methodology chart.

The research approach used for this investigation is depicted in Figure 2. The compilation of a database is the first step in developing a unique ANN-based model to forecast SC-2 transmission in a MM ventilated office setting using exhaled CO_2_ as a proxy indicator. The database preparation process consists of three major steps: (a) data collection; (b) data normalization; (c) data splitting for training, testing, and validation.

### 2.1. Data Collection

The data used in this study was collected in an MM ventilated office in India, utilizing a CO_2_ measurement instrument. In the year 2022, the measurements were made in the months of March, April, and May. The test site coordinates are NL 29°51′54″ and EL 77°54′10″. The office space has a floor area of 24 m^2^ and it has an 84 m^3^ volume. About 40% of the floor surface was taken up by furniture such as tables, chairs, and storage cabinets. The office is on the ground level and has a large window with an aluminum and glass frame that is 1.5 m in height and 2.5 m wide on the south wall. The window is split into three sections: the center section is fixed, while the sections at each end are operable and regarded as movable elements of the office. The main door and a side door of the office space are both made of wood. The front door, which measures 1.2 m × 2.4 m and leads to the passageway, is located on the north wall, right in the front of the window. The coupled ventilation above the main entrance door measures 1.2 m × 0.5 m in size. The office room’s east side wall contains the side door, which is 0.9 m × 2.0 m in size, and is regarded as a movable element of the office. A double door is used for the front door, while a single door is used for the side door. The room contains one ceiling fan. One window air conditioner is fitted under the fixed window pane at the height of 0.3 m from the ground floor. Table 3 outlines the additional room’s components in particular that are not mentioned in [33].

**Figure 3:**
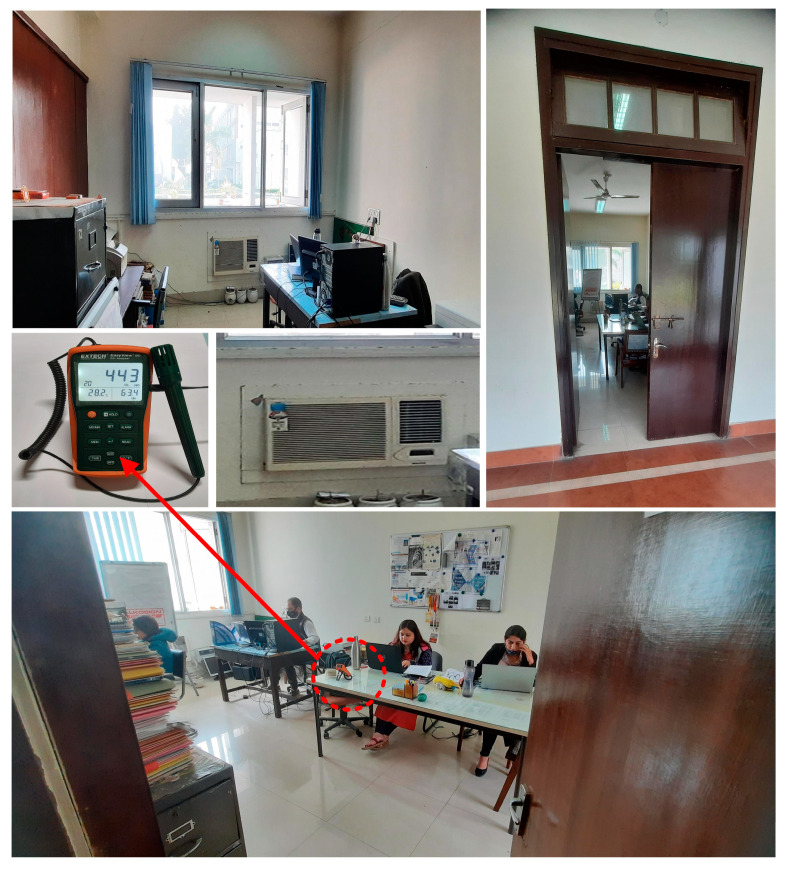
Real-time photographs of the observed indoor office setting.

Because the Hawthorne effect [57] would have an adverse influence on the participants’ typical behavior (talking, working, activity level, free motion, etc.), the study’s objective was kept confidential from both the dynamic and static occupants (subjects). If subjects knew they were being watched, their actions may have changed. If they were aware of the study objectives, they also may have altered their breathing patterns, movements, and other behaviors. However, only one person (the first author) was aware of the research goal, since they were collecting the information for this study. A previous [33] contained information on the subjects. As indicated in Figure 3, all of the static participants sat in the ‘L’ shape (longitudinal direction) at a space of around one meter.

**Figure 4:**
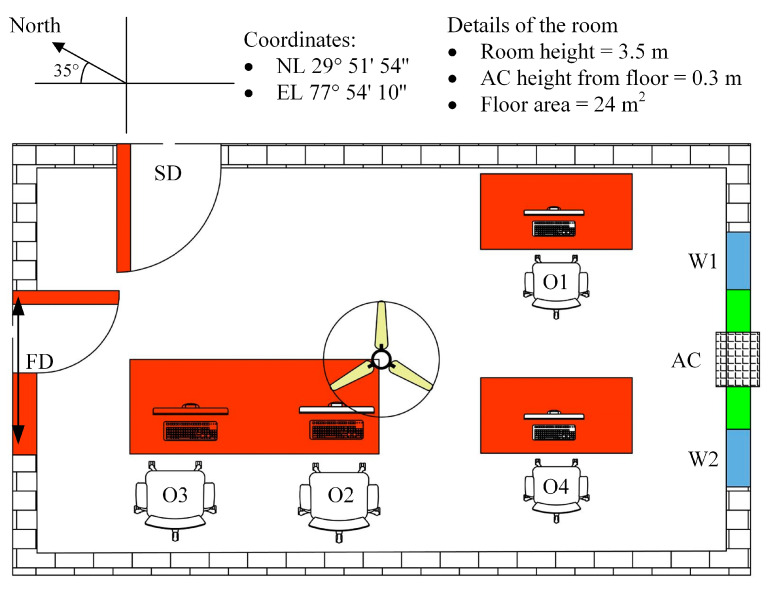
A 2-D representation of the mixed-mode ventilated office room.

**Figure 5:**
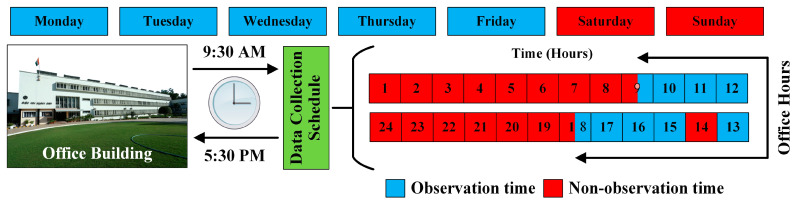
Monitoring schedule.

Figure 4 depicts a two-dimensional representation of the monitored office room. The different operational scenarios were based on the status of the ceiling fan, the status of the window air conditioner, and the operability of two doors and two windows. From Monday through Friday, data were collected 44 times each day at ten-minute intervals. Figure 5 depicts the data gathering schedule.

**Figure 6:**
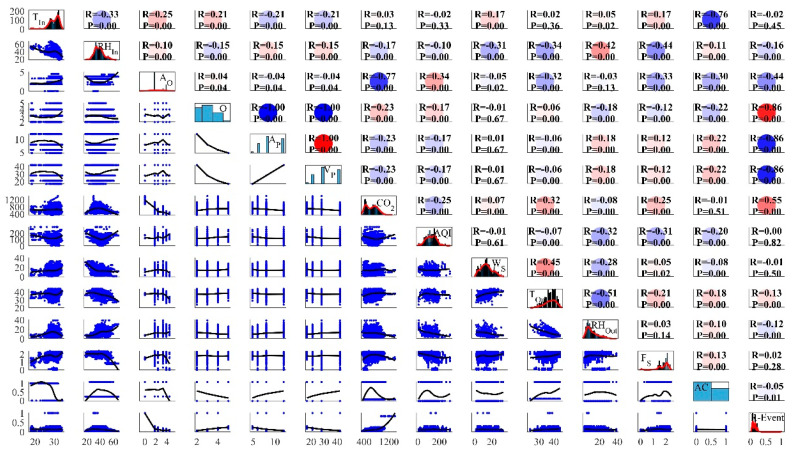
Extracted data scatter plot matrix.

The dataset plot matrix is presented in Figure 6. The two most crucial elements of a scatter plot diagram are the ‘*R*’ and ‘*P*’. The correlation coefficient between two values shows the fitting relationship for the selected datasets. The *p* value is the probability, which shows the relationship between the two values, which is equal to zero. Strong correlations have low *p*-values because the probability of the relationship parameters is very low. For ‘*P*’, the highest value is 0.82 and the lowest value is 0. ‘*R*’ is highest between the input features *A_P_* and *V_P_*, both of which have a value of one. However, the ‘*R*’ is minimal at points *A_P_* and *O*, as well as *V_P_* and *O*. At the aforementioned points, the minimal value of *R* is −1, as represented by the dark blue circles in the middle of Figure 6. 

The indoor temperature (*T_In_*), indoor humidity (*RH_In_*), area of opening (*A_O_*), number of occupants (*O*), area per person (*A_P_*), volume per person (*V_P_*), CO_2_ concentration (*CO_2_*), air quality index (*AQI*), outer wind speed (W_S_), outdoor temperature (*T_Out_*), outdoor humidity (*RH_Out_*), fan air speed (*F_S_*), and air conditioning (*AC*) were the thirteen input parameters that were collected. According to the input data, the CO_2_ concentration levels within the office room varied from 354 to 1398 ppm. The R-Event valued had a range of 0.04–1.00.

Table 4 presents the data for all of the input parameters and output parameters along with their minimum, maximum, and mean values. Additionally, the standard deviation, kurtosis, and skewness are also presented in the table. The mean indoor temperature was 29.84 °C, with a mean indoor relative humidity of 38.54 percent. The area of opening was 2.42 m^2^. The mean occupancy rate was 2.94. The mean *A_P_* and mean *V_P_* were 8.28 m^2^ and 30.90 m^3^, respectively. The mean CO_2_ concentration inside the office room was 648.42 ppm during the measurement period. The mean *AQI* was 124.94 and the outer mean wind speed was 13.97 kmph. The outdoor mean temperature and mean relative humidity were 37.43 °C and 11.95 percent. The mean values for the fan speed and air conditioning were 1.79 m/s and 0.40, respectively. The mean *R-Event* value for the MM case was 0.128, as presented in Table 4.

### 2.2. Standardization of Selected Data

After the collection of the datasets, the selected database for this study was standardized. The most crucial stage in defining data into definite ranges, such as 0 to 1, −1 to +1, 0 to +0.9, or 0 to +0.8, is the standardization stage [33,34,58]. The chosen input parameters’ values could change over a certain range. In order to make the computations simpler, data standardization was used. Equation (3) was used to standardize the data in this research study in the range of 0 to +0.8:

(3) $$N_{standardized}=0.8\times \frac{N-N_{min}}{N_{max}-N_{min}},$$

Here, *N* is the random value from the database, *N_standardized_* is the value that has to be normalized, *N_min_* is the parameter’s smallest value, and *N_max_* is its highest value.

### 2.3. Filtration of Data

In order to construct a multi-layered, feed-forward, back-propagation learning method, numerous researchers proposed using 70% of the data for training, 15% for validation, and 15% for testing. The data was divided into three portions with the 15:15:70 ratio of the entire dataset for testing, validation, and training purposes, respectively, following a short assessment of the ANN literature. After dividing the 2207 datasets, 331 datasets were utilized in this study for testing, 331 datasets for validation, and 1545 datasets for training.

## 3. R-Event Prognosis

In order to create the relationship and forecast the R-Event, two techniques were used in this study. The first technique was simply applying the first-degree order equation to the output and input parameters and was based on the CF method. The second approach was a type of artificial intelligence method, whereby supervised machine learning with multiple-variables ANN is applied to predict the R-Event for the mixed-mode ventilated office room.

### 3.1. Curve Fitting

**Figure 7:**
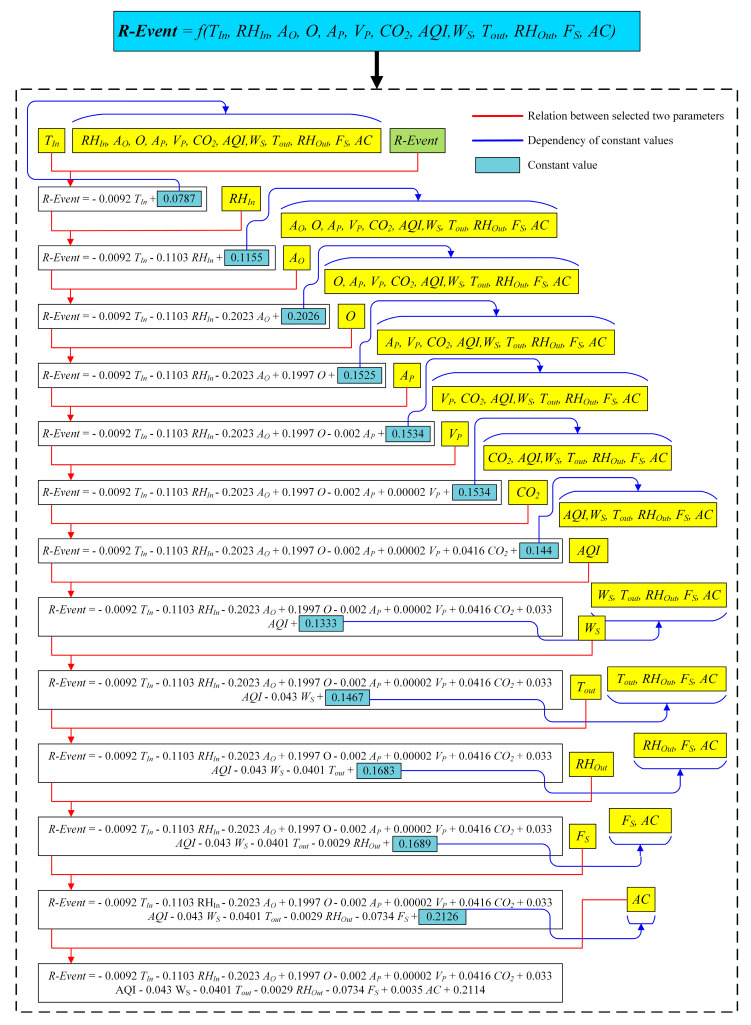
Curve fitting.

Figure 7 displays the curve fitting (CF) process applied in this work.

From Figure 7, let *N* = 0.2114.

The equation now becomes:

*R-Event* = −0.0092 *T_In_* − 0.1103 *RH_In_* − 0.2023 *A_O_* + 0.1997 *O* − 0.002 *A_P_* + 0.00002 *V_P_* + 0.0416 *CO_2_* + 0.033 *AQI* − 0.043 *W_S_* − 0.0401 *T_out_* − 0.0029 *RH_Out_* − 0.0734 *F_S_* + 0.0035 *AC* + *N*, (4)

The coefficient of correlation (*Cc*) may now be expressed computationally for all parameters as follows:

*C_c_* = −0.0092 *T_In_* − 0.1103 *RH_In_* − 0.2023 *A_O_* + 0.1997 *O* − 0.002 *A_P_* + 0.00002 *V_P_* + 0.0416 *CO_2_* + 0.033 *AQI* − 0.043 *W_S_* − 0.0401 *T_out_* − 0.0029 *RH_Out_* − 0.0734 *F_S_* + 0.0035 *AC*, (5)

Thus, the final equation can be expressed as follows:

*R-Event* = *C_c_* + *N*, (6)

### 3.2. Artificial Neural Networks

Frank Rosenblatt, a psychologist, developed the first ANN, also called a perceptron, in 1958, with the intention of simulating the human brain’s interpretations of visual information and object recognition process. Artificial neural networks have significantly aided in the implementation of several intelligent information processing techniques and the study of the fundamental functions of real neurons and brain activity, as well as in numerous industrial applications during the past forty years [59]. The ANN modeling method uses computers to mimic certain important features of the human nervous system, such as the ability to address problems by utilizing information from prior experiences in new situations.

Learning models such as ANNs construct a network of key connections between the selected target and considered features, which are connected by connections recognized as neurons and concealed behind layers. Each neuron contains a weight parameter as well as hidden or input connections to neurons from the layer above, each of which is weighted differently. The performance of the learning model depends significantly on the number of neurons in the hidden layer of the ANN. If the hidden layer’s population of neurons is too small, the error function will exhibit oscillating behavior and the network learning function will not be able to converge to an ideal value, making it difficult for the network to learn the relationships between the input–output configurations. When the number of neurons is too high, the input–output list is merely stored, resulting in poor generalization performance. The primary cause of overfitting is overtraining [60]. To achieve the best possible outcome, a trained ANN assembly can detect linkages between inputs and outputs by comparing measured and anticipated outputs [61].

Neural networks can be used to make models of difficult natural systems that have many inputs, making the models more accurate and simpler to use [62]. The most important feature in training is to minimize errors while increasing the value of *R*. This is achieved by modifying the weights during the course of the learning phase until the error function is achieved. In ANN, to evaluate the network performance, the ‘*R*’ and ‘*MSE*’ values are used [63]. Equations (7) and (8) express the ‘*R*’ and ‘*MSE*’ equations, respectively:

(7) $$R=\frac{\sum \left(x_{i}-\bar{x}\right)\times \left(y_{i}-\bar{y}\right)}{\sqrt{\sum {\left(x_{i}-\bar{x}\right)}^{2}\times \sum {\left(y_{i}-\bar{y}\right)}^{2}}\;\;},$$

*R* is the Pearson correlation coefficient, x_i_ represents the measured values in the datasets, the mean value of the measured values is $\bar{x}$, *y_i_* represents the predicted values in the datasets, and the mean value of the predicted values is $\bar{y}$.

(8) $$MES=\frac{1}{N}\sum_{i=1}^{N}{\left(x_{i}-y_{i}\right)}^{2},$$

The above is an iterative approach to changing the value of *w* by approximating *y_i_* and calculating the associated *MSE*. The errors are too large at first since the weights are chosen at random. The aim of network learning is to find the weights with the lowest level of error across all datasets. Approximating the weights using “trial and error” approaches would require a significant amount of effort as well as time. The gradient descent method is an effective way to quickly track the minimum sets of errors in a network training process. The gradient descent slopes down the error using the error gradient. In this work, an ANN model was suggested to forecast the *R-Event* value for the MM ventilated office room based on the observed conditions of the thirteen input features.

#### 3.2.1. ANN Modeling

**Figure 8:**
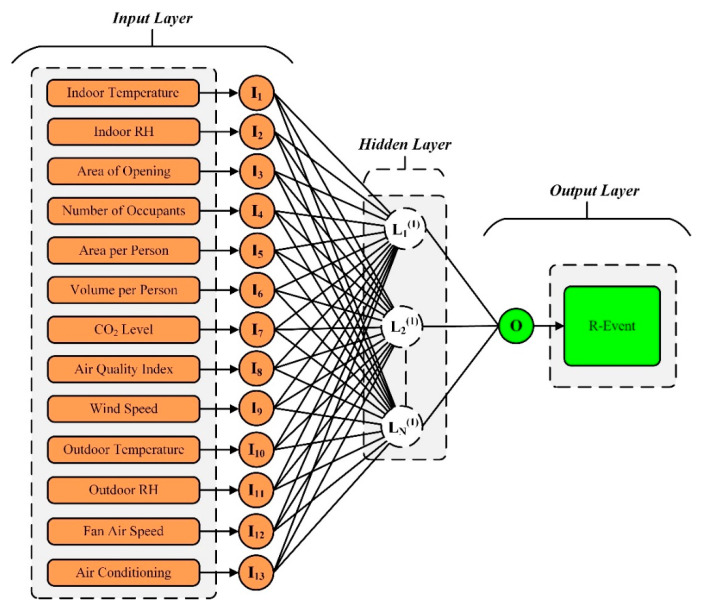
ANN architecture.

**Figure 9:**
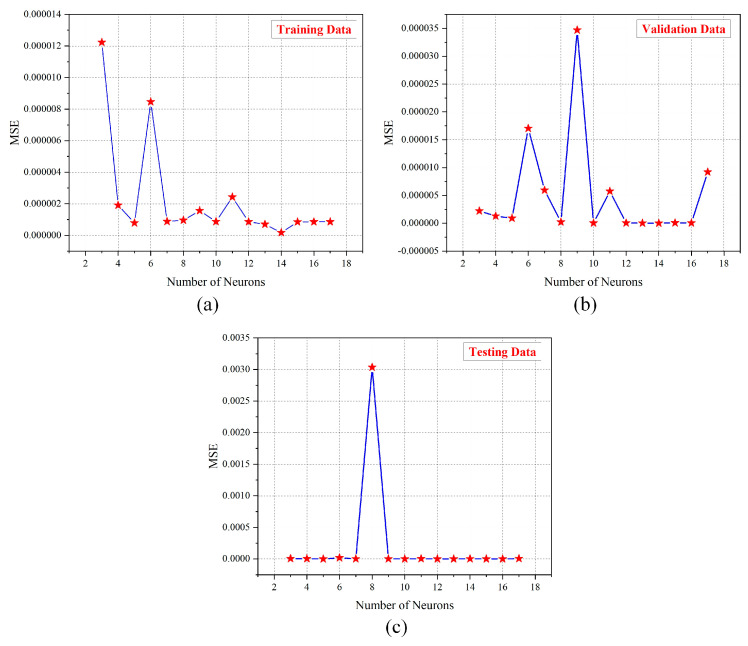
MSE plots of normalized values: (**a**) training; (**b**) validation; (**c**) testing.

**Figure 10:**
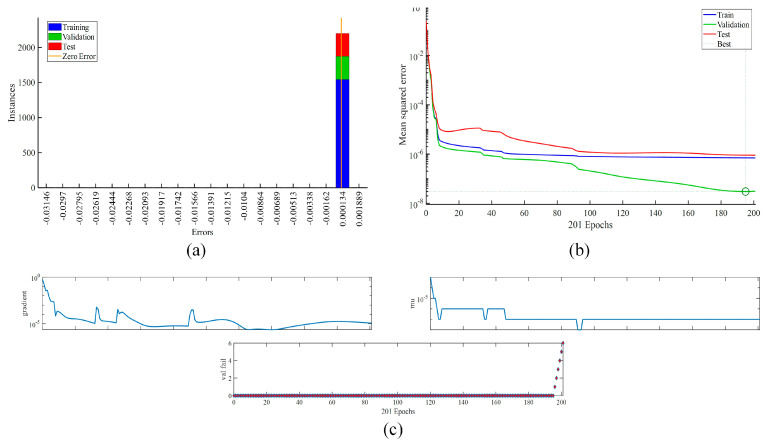
The ANN model’s performance results (13 neurons): (**a**) error plot; (**b**) training, validation, and testing data performance plot; (**c**) learning process.

Modeling is the procedure of describing a practical element or phenomenon as a series of computer assertions. It is critical to identify the network’s best architecture, providing both high precision and a well-fitted dataset. Figure 8 depicts the ANN architecture for this scenario, which includes thirteen input features and the R-Event as a target parameter. There is no method for calculating the precise number of hidden layers and neurons on every layer; thus, the number of ideal neurons and hidden layers was established via “trial and error”. After experimenting with several architectures and different counts of neurons in each layer, the ideal structure was discovered. The *MSE*-value-based network performance result is displayed in Figure 9. The model based on ANN started training from 3 neurons and trained until reaching 17 neurons. At the 3rd neuron the R of the training was 0.99916, while at the 17th neuron the R of training reached 0.99993. Each of the ANN models underwent at least 20 iterations. The suggested ANN model’s optimum design is illustrated in Figure 10. Each neuron’s *MSE* and *R* values were taken into consideration while determining its rank. In Table 5, the rankings of all neurons for their R and MSE values for training, testing, and validation are presented to show the overall best neurons. The 13th neuron trial had the lowest overall rating of all of the trials. The network that predicted the best performance among all neurons had a single hidden layer containing 13 neurons. The database features were linearly standardized in the range of 0 to 0.8 to speed up the learning process and promote quicker convergence. The ANN toolbox was used in the MATLAB (R2021a) environment to perform the simulations. The training techniques offered by MATLAB include Levenberg–Marquardt (LM), scaled conjugate gradient, and Bayesian regularization methods. The LM training method was chosen from among them due to its excellent convergence, high accuracy, and quick training of the network. This method often requires more memory but is quicker. The training ends when the generalization stops becoming better, as shown by an increase in the MSE of the validation samples. The same strategy was utilized in this study to randomly split the data into three portions: 15% for validation (331 datasets), 15% for testing (331 datasets), and 70% for training (1545 datasets). The activation functions for the hidden and output layers were selected to be TANSIG (Equation (9)) and PURELIN (Equation (10)), respectively:

(9) $$y=tangsig\left(x\right)=\frac{2}{\left(1+e^{-2x}\right)}-1,$$

(10) y=purlin(x)=x,

Figure 9 shows the *MSE* values for variable neurons for training, testing, and validation. The minimum *MSE* for the training is at the 14th neuron with a value of 0.00000070. The maximum *MSE* values for the training, testing, and validation are at the 3rd neuron, 8th neuron, and 9th neuron, with values of 0.00001223, 0.00303513, and 0.00003469, respectively. Table 6 presents the neuron ranking based on their *MSE* and *R* values.

For the MM ventilation datasets, Figure 10 shows the error and performance plots with the learning process. Figure 10b portrays the validation *MSE* as a green line, the training *MSE* as a blue line, and the testing *MSE* as a red line.

#### 3.2.2. Performance Indices

Basic performance indicators including the *R*, *RMSE*, *MAPE*, *MAE*, *NS*, and a20-index were taken into account while evaluating the effectiveness of the CF and ANN models in order to determine the models’ reliability [64,65,66,67,68]. It is clear that COVID-19 has affected many areas of our life, including our environment, activities, and other factors, and may require intelligent solutions using AI techniques, medical images, and clinical data to control the pandemic [69,70,71,72,73,74,75,76,77]. Formulas (11) to (16) below give the equations for the performance indices listed above. The following acronyms stand for the following terms: *R* is the Pearson correlation coefficient, *MAE* stands for the mean absolute error, *RMSE* for the root mean square error, *MAPE* for the mean absolute percentage error, and *NS* for the Nash–Sutcliffe efficiency index. Higher values of *R* and *NS* approaching 1 are used to measure a model’s accuracy. The most accurate model had values for the *MAE*, *RMSE*, and *MAPE* that were closest to zero. The most accurate model had the lowest values that were closest to zero for the *RMSE*, *MAE*, and *MAPE*. These performance indicators were used to assess how well the model could forecast *R-Event* values. In Section 3.2, Equations (7) and (8), corresponding to the *R* and *MSE* equations, are mentioned.

(11) $$MAE=\frac{1}{N}\;\sum_{I=1}^{N}\left|x_{i}-y_{i}\right|,$$

(12) $$MAPE=\frac{1}{N}\;\sum_{I=1}^{N}\left|\frac{x_{i}-y_{i}\;}{x_{i}}\right|\times 100,$$

(13) $$MSE=\frac{\sum_{i=1}^{N}{\left(x_{i}-y_{i}\right)}^{2}}{N},$$

(14) $$RMSE=\sqrt{\frac{\sum_{i=1}^{N}{\left(x_{i}-y_{i}\right)}^{2}}{N}},$$

(15) $$NS=1-\frac{\sum_{i=1}^{N}{\left(x_{i}-y_{i}\right)}^{2}}{\sum_{i=1}^{N}{\left(x_{i}-\bar{y_{i}}\right)}^{2}},$$

(16) $$a20-index=\frac{m20}{N},$$

The total number of values in the experimental dataset is denoted by *N*; $x_{i}$ and $y_{i}$ are the measured value and predicted value at *i*th level, respectively; $\bar{y_{i}}$ denotes the mean value of the predicted results.

## 4. Results and Discussion

**Figure 11:**
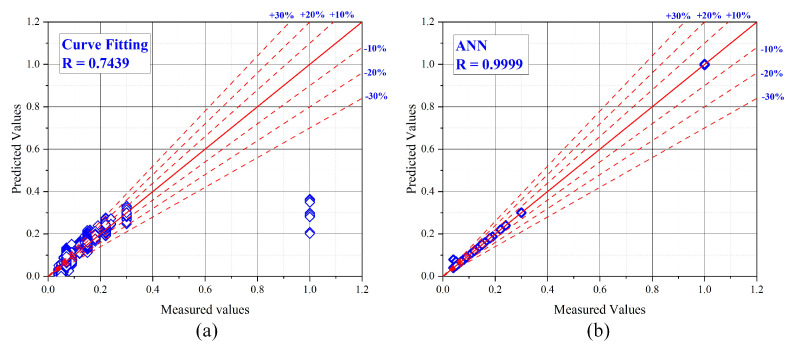
Comparison between the (**a**) CF model and (**b**) ANN model.

**Figure 12:**
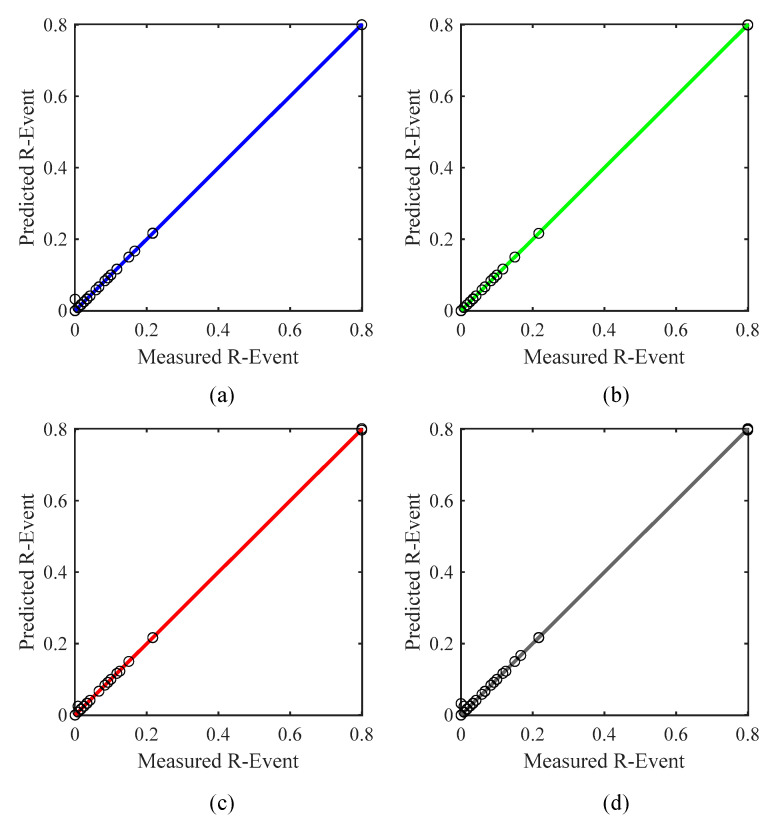
Correlation coefficients for the ANN model with the (**a**) training dataset, (**b**) validation dataset, (**c**) testing dataset, and (**d**) all datasets.

The *R* values for the CF and ANN models were 0.7439 and 0.9999, as shown in Figure 11a,b, respectively. The *R* value of the ANN model was 25.60% higher than the CF model. The other performance index values for the CF model, such as the *MAE*, *MAPE*, *RMSE*, *NS*, and a20-index values, were 0.0243, 23.2429, 0.0640, 0.5535, and 0.6611, respectively. Similarly, the performance index values for the ANN model, such as the *MAE*, *MAPE*, *RMSE*, *NS*, and a20-index values, were 0.0002, 0.1939, 0.0000, 0.9999, and 0.9991, respectively. The *MAE*, *MAPE*, and *RMSE* values for the CF model were 99.18%, 99.17%, and 100% higher than for the ANN model. The other performance factors such as the *NS* and a20-index values for the ANN model were 44.64% and 33.83% higher than for the CF model. As shown in Figure 11a, the plot of the CF model is more scattered as compared the ANN model. A correlation plot of the selected ANN model for training, validation, testing, and all datasets is presented in Figure 12. The results for the CF and ANN models are tabulated in Table 7. 

**Figure 13:**
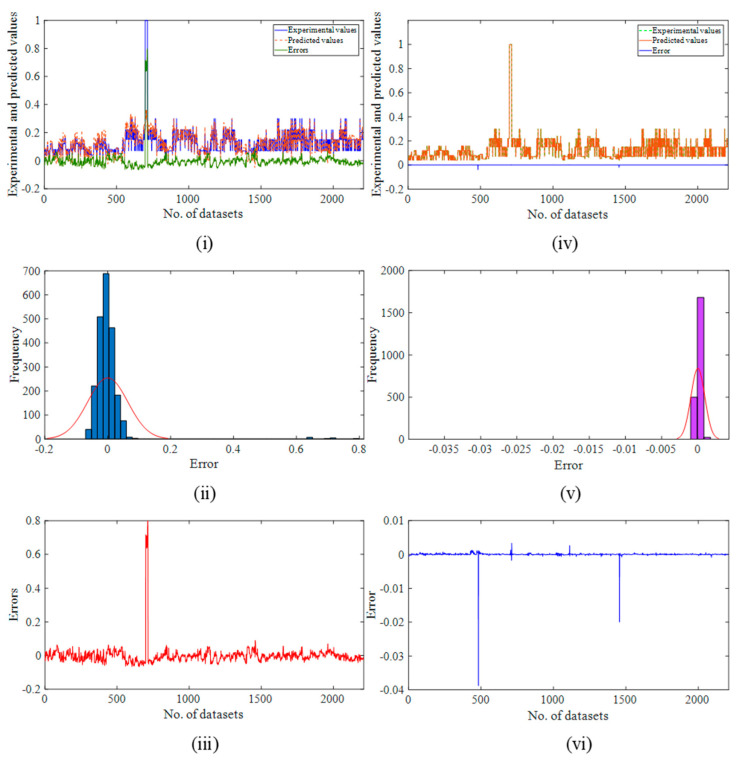
Results for the CF model and ANN model: (**i**,**iv**) variation in the predicted results; (**ii**,**v**) frequency distribution of the errors; (**iii**,**vi**) error plot with respect to the number of datasets.

The variation in the predicted results, frequency distribution of the errors, and error plot with respect to the number of datasets for the CF model are shown Figure 13i–iii, respectively. Similarly, in the ANN model, the aforementioned results are shown in Figure 13iv–vi, respectively. It is also clearly visible from the Figure 13 the more scattered results and errors observed for the CF model as compared to the ANN model. The predicted datasets in Figure 13vi are directly aligned with the actual values of the *R-Event*, and the blue line that depicts the errors is also straight. However, the CF model’s projected values and actual values are not quite in line, and the green line used to show this is similarly crooked. The histogram plot also confirms the reliability and accuracy of the ANN model. The constructed ANN model is more accurate and dependable than the CF model, which can be said after considering all of the performance parameters.

### ANN Formulation

The suggested ANN model displayed great accuracy and interpretability, as was already mentioned. The ANN model may directly yield the explicit formula for the *R-Event*. Equation (17) is the final equation to forecast the *R-Event*:

(17) $$R-Event=purlin\left(W_{HO}Y_{i}+B_{HO}\right)=W_{HO}Y_{i}+B_{HO},$$

Equation (18) illustrates the generalized formulation for the input to the hidden layer *Yi*.

(18) $$Y_{i}=W_{IH}N_{i,normalized}+B_{IH},$$

where *N_i_,_normalized_* represents the normalized inputs, *B_IH_* represents the biases between the input and hidden layers, *W_IH_* is the weight of the matrix in between the input and hidden layers, and *B_HO_* and *W_HO_* are the bias and weights of the hidden-to-output layer, respectively.

The proposed model is capable of predicting the R-Event for inputs with standardized projected data within the specified ranges. To forecast the indoor *R-Event* values for a mixed-mode ventilated office room scenario, the suggested model has thirteen parameters. The value of *Y_i_* can be obtained from Equation (20). The *R-Event* final equation is expressed in Equation (19):

(19) $$\begin{array}{r} R-Event=0.918042\;P1-0.000658\;P2-0.000418\;P3+0.071724\;P4-0.347533\;P5-0.000094\;P6+\\ 0.613483\;P7-0.000302\;P8+0.000570\;P9-0.154289\;P10-2.100065\;P11-0.001439\;P12-\\ 0.000447\;P13-0.922097, \end{array}$$

(20) $$\left[\begin{array}{l} P1\\ P2\\ P3\\ P4\\ P5\\ P6\\ P7\\ P8\\ P9\\ P10\\ P11\\ P12\\ P13 \end{array}\right]=tansig\left[\begin{array}{lllllllllllll} 0.0259 & 0.0172 & -4.876 & 0.5848 & 0.2421 & 0.5864 & 0.0419 & 0.0212 & -0.0021 & -0.0383 & -0.0306 & 0.0183 & 0.0118\\ -0.6037 & 0.1656 & 0.8141 & -0.3421 & -0.3894 & -0.8147 & -0.5389 & -0.1671 & -0.2992 & -0.3182 & 0.5486 & -0.0501 & -0.5349\\ -0.3236 & -1.1103 & 0.1577 & 0.6301 & 0.1844 & -0.4409 & 0.0465 & 0.0089 & 0.5931 & 0.3907 & -0.0286 & 0.4753 & -0.0682\\ 0.0785 & 0.3282 & -0.1912 & -0.3344 & 0.3654 & -0.8432 & -0.4074 & 0.0653 & 0.0047 & -0.0932 & -0.1859 & 0.1004 & -0.0179\\ -0.0533 & -0.0363 & 1.5260 & 0.0616 & 0.1306 & 0.8619 & -0.0343 & -0.0213 & -0.0077 & 0.0491 & 0.0435 & -0.0103 & -0.0169\\ 0.3224 & 0.5050 & 0.6743 & -1.0356 & -0.4815 & 0.0987 & -0.3502 & -0.4362 & -0.0676 & -0.1653 & -0.3669 & -0.1309 & -0.5452\\ 0.0433 & 0.0359 & -1.8143 & 0.1572 & 0.3415 & -1.5607 & 0.0226 & 0.0191 & 0.0054 & -0.0394 & -0.0385 & 0.0120 & 0.0131\\ -0.9313 & -0.0275 & 0.0662 & -0.1324 & 0.1394 & -0.3168 & -0.6585 & 0.0695 & 0.4311 & 0.3896 & -0.2283 & 0.8469 & 0.2990\\ -0.4282 & -0.0427 & -0.5763 & -0.2592 & 0.7416 & 0.7892 & -0.2553 & -0.0512 & -0.5749 & 0.7789 & -0.0993 & -0.0078 & 0.1542\\ -0.0466 & -0.0265 & 1.9032 & -0.7886 & 0.2820 & 0.3483 & -0.0664 & -0.0234 & -0.0091 & 0.0484 & 0.0394 & -0.0140 & -0.0184\\ 0.0177 & 0.0144 & -0.4158 & -0.0824 & 0.0610 & -0.3169 & 0.0085 & 0.0077 & 0.0022 & -0.0161 & -0.0157 & 0.0045 & 0.0054\\ -0.3941 & 0.0863 & 0.6681 & 0.6547 & 0.1347 & 0.6668 & 0.1728 & -0.5631 & 0.7191 & -0.2068 & 0.2249 & 0.3987 & 0.0584\\ -0.8408 & -0.1539 & 0.2897 & 0.4679 & -0.4228 & -0.3474 & 0.6702 & -0.6530 & -0.5026 & -0.0163 & -0.1091 & 0.4714 & -0.6776 \end{array}\right]\;\times \;\left[\begin{array}{c} T_{In}\\ RH_{In}\\ A_{O}\\ O\\ A_{P}\\ V_{P}\\ CO_{2}\\ AQI\\ W_{S}\\ T_{out}\\ RH_{Out}\\ F_{S}\\ AC \end{array}\right]+\left[\begin{array}{c} -3.9060\\ 1.3634\\ 1.2847\\ 1.5183\\ -0.0674\\ 0.1603\\ -1.4449\\ -0.2017\\ -0.5059\\ -2.4668\\ -0.7644\\ -1.5363\\ -1.5899 \end{array}\right]$$

In addition to creating CF- and ANN-based models, some additional broad observations were made throughout the investigation. A higher degree of human activity increases the likelihood of illness transmission and directly affects the CO_2_ content. For a safe working environment, the office size and volume are crucial factors. The occupancy rate, on the other hand, is the main variable that has a high correlation with the R-Event. Therefore, the occupancy level must be decreased to a safe level for a safe working environment. It is crucial to ventilate indoor settings to maintain low CO_2_ levels in order to stop the spread of SC-2. The CO_2_ level and indoor airborne transmission rate can be substantially correlated, demonstrating the need for ventilation. For improved operational control, maintaining all doors, windows, fans, and other ventilation systems is advised. Effective maintenance improves the effectiveness during full-scale operation. Nets are further advised for usage in order to stop insects from entering the office. Regular conversation is not encouraged in the workplace, and when it occurs without a mask, the risk of an infection spreading is increased. Installing CO_2_ sensors in offices to keep track of the CO_2_ levels is advised. The danger of viral transmission is considerably decreased via training and enforcing rules. 

## 5. Limitations of the Study

This study has several restrictions. This study’s focus is only on the interior spaces of the office buildings. Under an office setting working under a mixed-mode ventilation mode in a composite climatic environment, a singular source of infection is considered, with pathogen proliferation occurring exclusively through airborne transmission channels. A range of technological, financial, social, and chronological limitations affect this research. However, the procedure is consistent and may be used for a wide range of buildings in a wide range of climatic regions with a wide range of environmental conditions. During this investigation, the directions and patterns of air flow were not taken into account. However, they may significantly affect the transmission of airborne viruses, limiting the study’s applicability. Since office hours prevent the monitoring of nocturnal oscillations, only diurnal environmental fluctuations were taken into consideration. The participants were healthy adults with a normal breathing rate and no respiratory illnesses. The information acquired was case-based and reliant on several other environmental factors, such as the wind direction and pressure, which were not included in this study. This study considered all occupants occupying the office space without face masks and who were not immune to SC-2, thereby remaining susceptible. This study considered the delta variant of SC-2 for prediction purposes, which is estimated to be twice as infectious as the original virus. The numerous new and ancient SC-2 versions exhibit extremely inconsistent behavior. The interactions between humans and viruses are, therefore, not covered by this research. Human behaviors vary widely depending on a wide range of circumstances, which is a limitation in and of itself because each person has a certain behavioral pattern based on their experiences and brain prints. There were a few additional restrictions to this study, as the model could only predict results within the boundaries of the dataset. Furthermore, sneezing, coughing, and other respiratory activities were not considered in this study. People’s outer mobility (near office doors and windows) is also a significant component; however, owing to the extremely dynamic nature of such situations, this aspect was not taken into account in this study. The interference of outdoor CO_2_ concentrations was one more limitation of this study. The developed model predicts the average rate of event reproduction for an office room with diurnal variations, while other case-based alterations were limitations for the developed model. Close contact and fomite transmission were not considered. Full mixing with equal concentrations in the whole office room was considered, so the lack of non-uniform concentrations was one more limitation of the developed model. The models developed are single-zone models. Several parameters were uncertain, and were taken from the literature or estimated based on the current best available knowledge. The developed model was sensitive to the quanta emission rate values. The authors will address some of these limitations in future studies. More realistic and complex models can be built; however, the parametric uncertainty may still dominate the total uncertainty. Futuristic models can consider parameters based on the findings of new research to incorporate advanced knowledge.

## 6. Conclusions

AI-based modeling is usually a long and complex process. This study provides two models, one being an analytical model, i.e., the curve fitting (CF) model, and the second model being an AI-based model, i.e., the artificial neural network (ANN) model. Both the predictive models, namely the ANN and CF models, were tested and compared to forecast the R-Event values. The real-time data for the office environment were gathered in March, April, and May of 2022 in a mixed-mode (MM) ventilated office room situated in a composite climate. Thirteen features were used as inputs when developing the models. The R-Event was the target that was utilized as a proxy for the likelihood of SARS-CoV-2 infection transmission. The data were statistically examined first, then the CF model was used and the analyzed data were trained, tested, and validated with the ANN model. Only the ANN model did well in predicting the *R-Event* results when compared to the CF model. The ANN model showed a stronger correlation and lower error s(*R* = 0.9999, *MSE* = 0.0000, *RMSE* = 0.0000, *MAE* = 0.0002, *MAPE* = 0.1939, *NS* = 0.9999, and a20-index = 0.9991) with the measured results. When the data for the required input parameters are available, the ANN model may be utilized to reliably forecast the *R-Event*. The models created in this work may be used to forecast the *R-Event*, which serves as a proxy for estimating the likelihood that SARS-CoV-2 will be transmitted in MM office settings. As a result, time, effort, and human lives will be saved.

## Figures and Tables

**Table 1:** Specifications for the EXTECH Model EA80 instrument.

Veracity Factor	CO_2_	Air Temperature	Relative Humidity
Range	0~6000 ppm	−20~+60 °C	10~95%
Resolution	1 ppm	0.1 °C	0.1% RH
Accuracy	±3% of reading or ±50 ppm, whichever is greater @ 101.4 kPa @ 25 °C	±0.5 °C	±3% RH @ 25 °C, 30~95% RH ±5% RH @ 25 °C, 10~30% RH
Sensor type	Dual-wavelength detector with NDIR sensor	Thermistor	Precision capacitance sensor
Response time	<10 min	1 °C/2 s	45%→5% ≦ 1 min 95%→45% ≦ 3 min
Warm up time	10 s	-	-

**Table 2:** Specifications for the EXTECH Model HT200 instrument.

Veracity Factor	Air Temperature	Relative Humidity
Range	0~50.0 °C	1~99%
Resolution	0.1 °C	0.1%
Accuracy	±0.8 (@ 15~40 °C)	±3.0% RH (20~80%) ±5.0% RH (<20% or >80%)

**Table 3:** Additional components of the office room not listed elsewhere [33].

Component	Area/Volume	Fixed/Variable	Material
Ceiling Fan	-	Variable	Steel
Air Conditioner	-	Variable	Multiple Material

**Table 4:** Statistical analysis of the monitored parameters in the MM ventilated office environment.

Parameters	Symbol	Unit	Min.	Mean	Max.	Std.	Kurtosis	Skewness	Type
Indoor Temp.	*T_In_*	°C	17.6	29.8439	34.3	2.9229	6.8896	−1.6459	Inputs
Indoor RH	*RH_In_*	%	17.8	38.5449	67.5	7.4712	3.5377	0.4716	
Area of Opening	*A_O_*	m^2^	0	2.4208	4.5	0.9347	2.1744	0.7410	
Occupants	*O*	Nos.	2	2.9415	5	0.8277	2.2970	0.4161	
Area per person	*A_P_*	m^2^/person	4.8	8.8288	12	2.4564	1.5737	0.2497	
Volume per person	*V_P_*	m^3^/person	16.8	30.9009	42	8.5976	1.5737	0.2497	
CO_2_ Level Inside	*CO_2_*	ppm	354	648.4232	1398	177.0212	2.3827	0.3235	
*AQI*	*AQI*	-	7	124.9393	299	49.3736	3.2166	0.1602	
Wind Speed	*W_S_*	km/h	1.4	13.9738	33.6	6.9065	2.5846	0.3812	
Outdoor Temp.	*T_out_*	°C	24	37.4300	44	3.8624	2.9119	−0.6020	
Outdoor RH	*RH_Out_*	%	3	11.9515	39	6.7152	3.6486	1.0699	
Fan Speed	*F_S_*	m/s	0	1.7850	2.4	0.4544	7.3613	−1.8744	
*AC* Operation	*AC*	-	0	0.3960	1	0.4892	1.1808	0.4252	
*R-Event*	*R*-*Event*	-	0.04	0.1280	1.00	0.0957	50.3785	5.5647	Output

**Table 5:** Selection of the best neuron based on the ‘*R*’ and ‘*MSE’* values.

S. No.	Neuron	Statistical Parameters					
		*R*			*MSE*		
		Training	Validation	Testing	Training	Validation	Testing
1	3	0.999163	0.999571	0.999499	0.00001223	0.00000221	0.00000699
2	4	0.999856	0.999838	0.999749	0.00000191	0.00000131	0.00000446
3	5	0.999937	0.999937	0.99999	0.00000079	0.00000091	0.00000011
4	6	0.99948	0.999215	0.999442	0.00000846	0.00001705	0.00001795
5	7	0.99993	0.999531	0.999844	0.00000088	0.00000594	0.00000236
6	8	0.999923	0.999975	0.81034	0.00000096	0.00000021	0.00303513
7	9	0.999882	0.998693	0.999804	0.00000156	0.00003469	0.00000252
8	10	0.999927	0.999998	0.999997	0.00000087	0.00000004	0.00000002
9	11	0.999771	0.999678	0.999773	0.00000243	0.00000575	0.00000433
10	12	0.999922	0.999995	0.999996	0.00000086	0.00000005	0.00000008
11	13	0.999946	0.999997	0.999926	0.00000070	0.00000003	0.00000090
12	14	0.999985	0.999997	0.999861	0.00000017	0.00000001	0.00000336
13	15	0.999932	0.999994	0.999989	0.00000085	0.00000008	0.00000012
14	16	0.999938	0.999995	0.999992	0.00000085	0.00000006	0.00000008
15	17	0.999931	0.999483	0.999549	0.00000086	0.00000921	0.00000681

**Table 6:** Rankings for the best neuron.

S. No.	Neuron	Ranking						Ranking Score	Ranking
		*R*			*MSE*				
		Training	Validation	Testing	Training	Validation	Testing		
1	3	15	11	13	15	10	13	77	14
2	4	12	9	11	12	9	11	64	11
3	5	4	8	4	3	8	4	31	6
4	6	14	14	14	14	14	14	84	15
5	7	7	12	8	9	12	7	55	8
6	8	9	7	15	10	7	15	63	10
7	9	11	15	9	11	15	8	69	13
8	10	8	1	1	8	3	1	22	3
9	11	13	10	10	13	11	10	67	12
10	12	10	5	2	6	4	2	29	5
11	13	2	2	6	2	2	6	20	1
12	14	1	3	7	1	1	9	22	2
13	15	5	6	5	4	6	5	31	7
14	16	3	4	3	5	5	3	23	4
15	17	6	13	12	7	13	12	63	9

**Table 7:** Comparison of the performance indicators for the CF and ANN models.

Proposed Model	Performance Indicators						
	*R*	*MAE*	*MAPE*	*MSE*	*RMSE*	*NS*	a20-index
CF	0.7439	0.0243	23.2429	0.0041	0.0640	0.5535	0.6611
ANN	0.9999	0.0002	0.1939	0.0000	0.0000	0.9999	0.9991

## Data Availability

The data will be shared upon reasonable request.
